# Ultralow-angle faults produce giant earthquakes

**DOI:** 10.1126/sciadv.aee3921

**Published:** 2026-07-01

**Authors:** Satoshi Ide, Mikito Furuichi, Dye S. K. Sato

**Affiliations:** ^1^ Department of Earth and Planetary Science, The University of Tokyo, Tokyo, Japan.; ^2^ Research Institute for Value-Added-Information Generation, Japan Agency for Marine-Earth Science and Technology, Yokohama, Japan.; ^3^ Research Institute for Marine Geodynamics, Japan Agency for Marine-Earth Science and Technology, Yokohama, Japan.

## Abstract

What controls the occurrence of giant earthquakes remains a central question in seismology. We reveal a notably simple—but previously overlooked—relationship between earthquake size and fault dip in subduction zones. Global earthquake statistics show a robust dependence of earthquake size on fault dip, with the probability of extreme events peaking on ultralow-angle faults. Through three case studies, we demonstrate that earthquakes tend to grow larger when the fault geometry is aligned with the regional stress field. We further discuss how such favorable stress configurations are generated along subduction interfaces and emphasize the critical importance of monitoring the temporal evolution of stress in subduction zones to better forecast future megaearthquakes.

## Teaser

In subduction zones, low-angle faults are better aligned with the stress field and more prone to large earthquakes.

## INTRODUCTION

Extremely large earthquakes such as the 2004 Sumatra, Indonesia (*M*_w_: 9.3), the 2010 Maule, Chile (*M*_w_: 8.8), and the 2011 Tohoku-Oki, Japan (*M*_w_: 9.1) earthquakes predominantly occur in subduction zones along thrust-type plate boundaries. A common explanation for such large earthquakes is that the subduction of a cold oceanic plate lowers temperatures along the plate interface, promoting brittle behavior in rocks and allowing rupture to propagate over a wide area (*1*). Naturally, large earthquakes need large faults. However, a large fault is only a necessary condition: A single event does not necessarily rupture the entire fault—strain may instead be released through numerous smaller earthquakes without producing a giant one.

Another key aspect of large earthquake occurrence is the relationship between earthquake size and frequency, described by the Gutenberg-Richter law (*2*)

log10N=a−bM (1)

where N is the number of earthquakes with magnitude ≥M and a and b are constants. The b value controls the ratio of small to large earthquakes. Recent studies suggest that earthquake rupture initiates at scales below observational resolution and grows in a self-similar manner, making it impossible to predict the final size of an event (*3*, *4*). Therefore, the b value also reflects the likelihood of rupture growth. A smaller b value indicates a higher probability of rupture growth and more large earthquakes, while a larger b value suggests that small events dominate. When b>1.5, most seismic moment is released by small earthquakes. In many active regions, the b value is close to 1, meaning that a single large earthquake releases a substantial portion of the total seismic moment.

However, even when the b value is close to 1, a small difference can lead to a large difference in the probability of a giant earthquake. For example, the probability that a small *M* 2 earthquake—common in subduction zones—will grow into a *M* 9 event is more than 600 times higher when b=0.8 than when b=1.2. If earthquakes are a probabilistic process that grows in a self-similar manner, then the likelihood of actually experiencing a giant earthquake depends sensitively on such differences in the b value. Although numerous studies have investigated why the largest earthquakes occur in subduction zones (*5*–*7*), differences in probability arising from variations in the b value have not been explicitly considered. In this study, we therefore examine the mechanisms of giant earthquake occurrence with a primary focus on the b value.

## RESULTS

### Rupture growth probability and stress state

The b value is believed to vary with the stress state (*8*). Laboratory experiments show that increasing differential stress—defined as the difference between the maximum and minimum principal stresses ($\text{Δσ}=\sigma_{1}-\sigma_{3}$)—leads to lower b values (*9*, *10*). For inland earthquakes, b value tends to increase with depth, consistent with increasing lithostatic pressure (*11*, *12*). In subduction zones, spatial variations in the *b* value have been linked to changes in stress regime associated with differences in plate ages (*13*). These variations in the b value may therefore provide a means to assess the likelihood of future large earthquakes (*14*, *15*).

The b value also depends on faulting style (*16*). b values estimated from the Global CMT (GCMT) catalog (*17*) are about 0.2 lower for thrust faults than for normal faults, indicating that small earthquakes on thrust faults are more likely to grow into larger events. This difference reflects variations in differential stress and can be explained using Anderson’s theory (*18*) together with the Coulomb failure criterion. Near Earth’s surface, one of the principal stress axes is vertical due to the traction-free boundary condition. In normal faulting, the $\sigma_{1}$ axis is vertical, whereas in thrust faulting, the $\sigma_{3}$ axis is vertical and the $\sigma_{1}$ axis is horizontal. Because vertical stress is determined by the weight of the overlying material, it is similar in both cases. If $\sigma_{3}$ in thrust faulting equals $\sigma_{1}$ in normal faulting, the average compressive stress, $\left(\sigma_{1}+\sigma_{3}\right)/2$, is higher for thrust faults. Assuming the shear stress (τ), normal stress ($\sigma_{n}$), and pore pressure (p) on the fault plane obey the Coulomb failure criterion

$$\tau =\mu \left(\sigma_{n}-p\right)$$ (2)

and the coefficient of friction μ is the same, thrust faults experience higher shear and differential stresses than normal faults—explaining their lower *b* values.

Anderson’s theory and the Coulomb failure criterion also account for fault orientation. Normal faults tend to have steep dip angles, whereas thrust faults generally dip at lower angles. According to the Coulomb criterion, the differential stress required for failure depends on the angle θ between the fault plane and the $\sigma_{1}$ axis. The optimal failure angle $\theta^{\text{opt}}$ is less than 45°. For typical values of the friction coefficient μ= 0.6 to 0.85 (*19*), $\theta^{\text{opt}}$ ranges from 24.8° to 29.5°. For lower friction (μ = 0.1 to 0.2), $\theta^{\text{opt}}$ increases to 39.3° to 42.1°. Thus, when the $\sigma_{1}$ axis is vertical (normal faulting), failure occurs on steep (50° to 60°) faults, while a horizontal $\sigma_{1}$ axis favors low-angle (30° to 40°) faults.

Thus, many subduction-zone earthquakes occur on low-angle faults, and their particularly low b values help explain the frequent occurrence of large events. However, past extremely large earthquakes have ruptured fault planes dipping well below 30°. The dip angles of the 2004 Sumatra, 2010 Maule, and 2011 Tohoku-Oki earthquakes are 8°, 18°, and 10°, respectively (GCMT). The corresponding dip angles of the plate interfaces are also mostly within 10° to 20° (*6*, *20*). What makes these ultralow-angle faults capable of hosting such massive earthquakes?

### Dip dependence of rupture growth probability

**Fig. 1.**
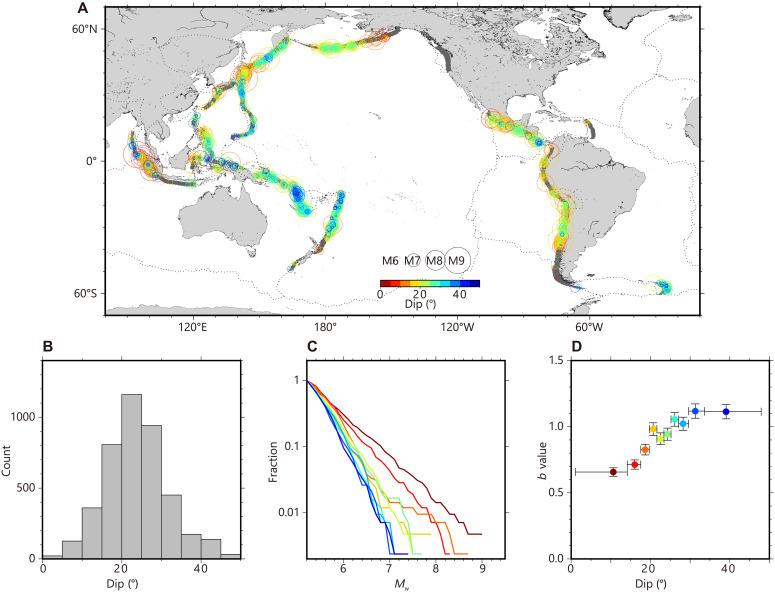
Dip angle and *b* value of shallow reverse-fault earthquakes. (**A**) Spatial distribution of analyzed earthquakes. Events are from the 1976–2024 GCMT catalog, shallower than 30 km and with *M*_w_ > 5.2, and classified as reverse faults (both nodal planes have rake angles between 60° and 120°). Circle size reflects magnitude, and color indicates dip angle. Dashed lines show plate boundaries from PB2002 (*19*). Gray shaded regions mark areas within 200 km landward of the trench axis (*20*). (**B**) Histogram of dip angles. (**C**) Magnitude-frequency distributions for 10 dip-angle bins, with colors corresponding to the ranges shown in (D). (**D**) Relationship between dip angle and *b* value. Horizontal bars represent the dip range of each bin, and vertical bars indicate the SE.

Earthquakes on ultralow-angle faults exhibit low b values. Using the 1976–2024 GCMT catalog, we selected shallow (<30-km depth) subduction-zone thrust earthquakes based on the PB2002 plate model (*21*, *22*) and grouped them according to the dip angle of the lower-angle nodal plane. The b value and its SE were calculated for each group using the maximum-likelihood method (Materials and Methods, Fig. 1, and fig. S1) (*23*). A clear trend emerges: The b value is approximately 1.1 for dips greater than 30°, decreasing to about 0.65 for dips shallower than 15°. The small SE (~0.05) confirms that the trend is statistically significant. This relationship is observed regardless of whether the earthquakes are mainshocks (isolated) or aftershocks (triggered), as classified using the nearest neighbor clustering method (fig. S2) (*24*). A similar trend is not observed for deep events (fig. S3).

A b value of 0.65 is extremely low. Interpreting the b value as the likelihood that a small earthquake grows into a large one, such a low value suggests that ruptures on ultralow-angle faults are especially prone to grow large. This finding has critical implications for seismic hazard assessment. Could this unusually low b value also reflect high differential stress? If the $\sigma_{1}$ axis is horizontal, thrust faults dipping below 20° are misoriented relative to the stress field and may require a greater differential stress to rupture. It is therefore plausible that misoriented faults tend to have lower b values and are more susceptible to rupture growth. While counterintuitive, this hypothesis remains largely untested. Below, we examine the relationship between fault orientation, the background stress field, and b values.

### Effect of fault and stress orientations

**Fig. 2.**
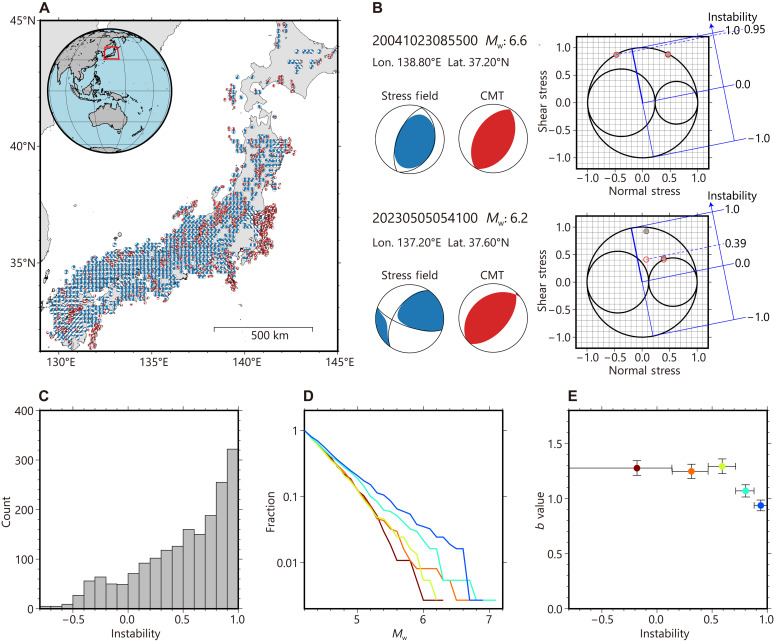
Relationship among estimated stress field, earthquake mechanisms, and *b* values in Japan. (**A**) Locations of stress field estimates and earthquake distribution. The estimated stress orientations are shown as blue beachballs representing the deviatoric stress tensor at each location. Earthquake focal mechanisms (NIED F-net CMT solutions, depth < 30 km, *M*_w_ > 4.2) are shown as red beachballs. The inset map indicates the study area. (**B**) Explanation of Mohr circles and instability. For two selected earthquakes, the local stress field (blue) and CMT solutions (red) are illustrated. The right panel shows Mohr circles normalized by differential stress. The two small black circles mark the maximum shear stress on the nodal planes, and the two red circles show the corresponding shear stress in the slip directions. The method for calculating instability is illustrated in the blue stress coordinate system. Lon., longitude; Lat., latitude. (**C**) Histogram of instability values. (**D**) Magnitude-frequency distributions, computed after grouping events into five bins based on instability. Colors correspond to the instability ranges shown in (E). (**E**) Relationship between instability and *b* value. Horizontal bars indicate the data range within each bin; vertical bars show SEs.

We first examined earthquakes in Japan. Using the inland stress map (*25*) and focal mechanism solutions from the National Research Institute for Earth Science and Disaster Resilience (NIED) F-net catalog, we evaluated the consistency between each earthquake and the regional stress field (Fig. 2A and Materials and Methods). We adopted the fault instability index (*26*), which is slightly modified from the original definition to be 1 for optimally oriented rupture (at $\theta^{\text{opt}}$) and −1 for least favorable orientation (Fig. 2B). More accurately, it is the inner product between (i) the vector from the origin to an arbitrary stress state within the Mohr circle normalized to unit radius and (ii) the vector from the origin to the tangency point between the Mohr circle and the Coulomb failure criterion. For each event, we computed the instability for both nodal planes and used the larger value. We analyzed shallow (<30 km) earthquakes with *M*_w_ ≥ 4.2, within 0.15° of a stress estimate. Most instability values cluster near 1 (Fig. 2C), confirming that focal mechanisms generally align with the stress field. Grouping events by instability reveals a clear decrease in b value with increasing instability (Fig. 2, D and E). That is, more misoriented faults are less likely to host large earthquakes. This trend depends on the assumed friction μ, with a monotonic decrease observed for 0.1<μ<0.4. A similar pattern has been reported for seismicity for the Western Tottori region (*27*).

**Fig. 3.**
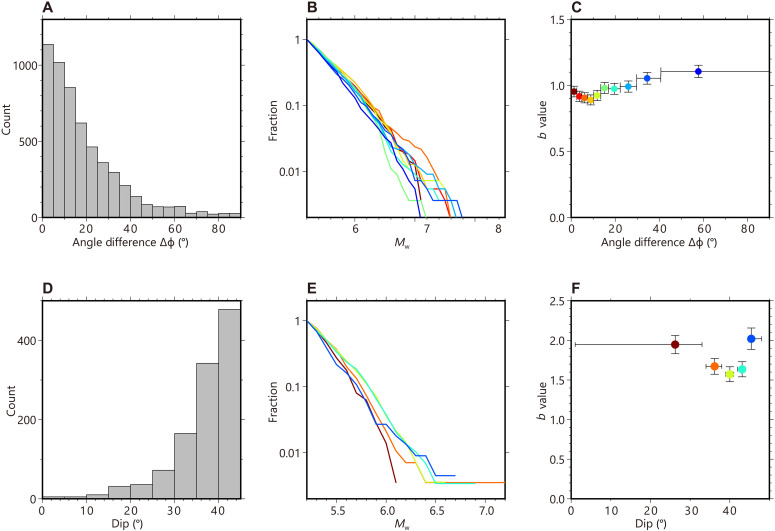
Relationship between focal mechanism–stress field consistency and *b* value using the GCMT catalog. (**A** to **C**) Comparison of the estimated global maximum horizontal compressive stress direction (*SH*_max_) and the *P* axis of focal mechanisms, expressed as the angular difference Δϕ. (A) Frequency distribution of Δϕ. (B) Magnitude-frequency statistics, with color indicating Δϕ ranges shown in (C). (C) Relationship between Δϕ and *b* value. Data are divided into 10 bins with equal event counts. Horizontal error bars indicate the Δϕ ranges of each bin; vertical error bars represent the SEs. (**D** to **F**) Relationship between the dip of the lower-angle nodal plane and *b* value for normal-faulting earthquakes near extensional plate boundaries worldwide. (D) Histogram of dip angles. (E) Cumulative magnitude-frequency distributions, with colors indicating dip angle ranges shown in (F). (F) Relationship between dip angle and *b* value. Horizontal bars show dip angle ranges for each bin; vertical bars represent the SEs.

The global stress field has also been estimated. The World Stress Map (WSM) (*28*) provides estimates of the maximum horizontal compressive stress (*SH*_max_) direction worldwide. We analyzed strike-slip earthquakes (Materials and Methods), selecting events in which both nodal planes dip at angles greater than 60°, and measured the angle Δϕ between the *P* axis of the focal mechanism and *SH*_max_ direction (fig. S4). Most Δϕ values are small (Fig. 3A), although some exceed 45°, implying substantial misalignment (fig. S5). A clear trend between Δϕ and b value emerges: Events with smaller Δϕ exhibit lower b values. Notably, the minimum b value (~0.9) occurs not at Δϕ = 0° but around 10°. If μ = 0, then the optimal failure angle $\theta^{\text{opt}}$ = 45°, and the *P* axis aligns with *SH*_max_. The observed offset therefore suggests μ > 0. For μ = 0.1, 0.2, and 0.4, the expected offsets are 2.9°, 5.7°, and 10.9°, respectively. For Δϕ > 40, the b values approach ~1.1. With the SEs less than 0.05, the ~0.2 difference of b value is statistically significant, confirming that ruptures misaligned with the stress field are less likely to grow into large earthquakes.

Because both the Japanese stress map and WSM are derived from focal mechanisms, they are not independent of the events they describe. To avoid this bias, we examined settings where the stress orientation can be reasonably inferred. At mid-ocean ridges, the $\sigma_{1}$ axis is assumed to be vertical and the $\sigma_{3}$ axis horizontal. On the basis of this assumption, we analyzed normal faulting earthquakes within 100 km of ridge axes defined by the PB2002 plate model (fig. S6) (*21*). Events grouped by the dip of the shallower nodal plane cluster around 45° (Fig. 3D). Dividing them into five dip-angle groups, we found that the b value reaches a minimum near 40° (Fig. 3, E and F). Although overall b values are high—indicating that deformation is largely accommodated by small events—ruptures at optimal angles are still more likely to grow. Furthermore, the aforementioned study on rake dependence of b values (*16*) presented smoothed b value variation. Without smoothing, however, local minima emerge near the angles predicted by Anderson’s theory (0°, ±90°, and ±180°) (fig. S7). This supports the idea that b values are strongly controlled by compatibility with the background stress field.

While factors such as differential stress (*8*), material heterogeneity (*29*), and the fractal dimension of fault structures (*30*) have been proposed to explain variations in b values, our results suggest that the alignment between focal mechanisms and the background stress field is also a key factor. Earthquakes that are well aligned with the stress field are more likely to grow, resulting in smaller b values—conditions favorable for very large ruptures. In a uniform stress field, the minimum b value likely corresponds to the optimally oriented plane with the highest Coulomb failure stress (CFS), suggesting that b values reflect local CFS conditions (*9*, *29*). Moreover, the lowest b values occur under stress conditions equivalent to a friction coefficient μ ≈ 0.2, implying that the effective friction along plate boundaries may be substantially lower than the ~0.6 typically observed in laboratory rock experiments, as suggested by previous research (*31*–*33*).

## DISCUSSION

**Fig. 4.**
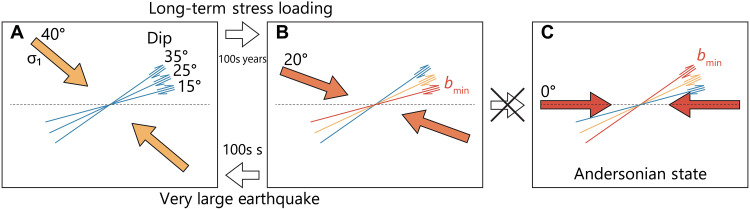
Changes in stress state along plate boundaries and the likelihood of reverse faulting at various angles. (**A**) Stress state when accumulated elastic energy along the plate interface is at a minimum. None of the fault orientations are close to the optimal angle for rupture. (**B**) Stress increases through long-term loading, and the orientation of ultralow-angle faults approaches the optimal angle. A megathrust earthquake releases the accumulated stress, returning the system to the state shown in (A). (**C**) Idealized Andersonian state with a horizontal maximum principal compressive stress—a condition that is never realized in subduction zones.

With these findings in mind, we return to the dip dependence of the b value in subduction zones. In reality, the $\sigma_{1}$ axis is not horizontal; in many subduction zones, it dips seaward by approximately 20° to 40° (*34*), favoring rupture on thrust faults dipping 10° to 20°. Observations show that the $\sigma_{1}$ axis rotates from near horizontal before megathrust events to a steeper angle afterward (*35*, *36*). If such a stress cycle recurs (Fig. 4), then the plunge of the $\sigma_{1}$ axis decreases from ~40° to ~20° and then recovers to ~40° after the largest event (Fig. 4, A and B). Therefore, the purely horizontal compression assumed by Anderson’s theory (Fig. 4C) is never realized. During this cycle, the very-low–angle fault (~15°) remains closer to optimal than faults dipping ~30°, which would be near optimal only under purely horizontal compression. Just before the largest event (Fig. 4B), the shallow $\sigma_{1}$ axis renders ultralow-angle faults nearly optimal for rupture, explaining why such faults host the largest earthquakes with a high probability—that is, with a small b value.

Assuming that rupture occurs on optimally oriented planes, the plunge angle δ of the $\sigma_{1}$ axis, differential stress Δσ, vertical stress $\sigma_{\mathrm{zz}}$, pore pressure p, and friction coefficient μ are related by (Materials and Methods and fig. S8)

$$\text{Δσ}=\frac{2\left(\sigma_{\mathrm{zz}}-p\right)}{\frac{\sqrt{1+\mu^{2}}}{\mu }-\text{cos}2\delta }$$ (3)

This relation shows that lower plunge angles correspond to higher differential stress. Although this behavior is expected from tectonic loading, it implies that stress peaks just before a megathrust event (Fig. 4B). If b values reflect differential stress, this framework naturally explains the observed low b values on very-low–angle (~15°) thrust faults. In contrast, on faults dipping ~30°, this stress is far from optimal, and b values cannot be low even with a high differential stress. Because earthquakes with dip angle around 30° are frequent (Fig. 1B), these faults may have a higher probability of rupture at smaller scales. However, such ruptures often fail to grow because of misalignment with the regional stress field at larger scales. A full understanding of earthquake nucleation and arrest therefore requires consideration of not only average stress state but also its spatial heterogeneity across scales.

We conclude that ultralow-angle faults not only provide a large fault area but also host earthquakes that are more prone to growth because their orientations are closer to optimal under conditions of large differential stress. However, this explanation is not necessarily unique and does not exclude other possible interpretations. This finding may contribute to a more accurate understanding of the mechanisms governing the occurrence of the largest megathrust earthquakes.

As demonstrated across various earthquake types, the b value reflects not only regional differential stress but also the alignment between the stress field and fault orientation. This finding calls for reinterpreting past observations. For example, the depth dependence of b value (*11*, *12*) may result from both increasing stress and changing fault alignment with depth. Postseismic increases in b value (*14*) not only likely reflect stress drop near the rupture but may also involve temporal variations in stress orientation. The decrease in b value observed before the Tohoku-Oki earthquake (*15*) may indicate that the preparation of stress conditions favored rupture growth along the plate interface. These insights underscore the importance of mapping stress fields and monitoring b values for seismic hazard assessment.

## MATERIALS AND METHODS

### Analysis for subduction-zone thrust fault earthquakes

We use the GCMT catalog (*14*) covering the period from 1976 to 2024. Following the PB2002 plate boundary model (*19*), subduction zones were defined as regions extending 200 km landward from the trench axis along the plate-convergence direction (*20*). We extract earthquakes shallower than 30 km that occurred within these regions and whose two nodal planes both had rake angles between 60° and 120° (fig. S1). For each event, the nodal plane with the smaller dip was selected, and the events were divided into 10 groups based on dip angle. For each group, the *b* value and its SE were estimated. The magnitude of completeness ($M_{c}$) was tested over the range of 5.2 to 5.5 to ensure that the results were stable. To confirm that the results were not biased by a limited number of events, we repeated the same analysis for subcatalogs divided into 12-year intervals beginning in 1976. Although variability increased, the same general trend was observed (fig. S1).

### Estimation of *b* value

Assuming that all earthquakes above a given completeness magnitude ($M_{c}$) were detected within each group, we estimated the b value of the Gutenberg-Richter law using the maximum likelihood method (*21*). For N earthquakes with magnitudes $M_{1},M_{2},\ldots ,M_{N}$, the b value is given by

$$b=\frac{{\text{log}}_{10}e}{\bar{M}-M_{c}}$$

where $\bar{M}$ is the average magnitude of the N events. The SE of the b value was calculated as

$$\sigma_{b}=\frac{b}{\sqrt{N}}$$

### Focal mechanisms and background stress in Japan

The stress field in the shallow inland region of Japan (<20-km depth) was estimated (*22*) as deviatoric stress tensor on a 0.2° grid, using assumptions similar to those of Michael (*34*, *35*). Some inland areas lack reliable stress estimates. For earthquakes, we used centroid moment tensor (CMT) solutions from the F-net catalog provided by the NIED. We selected events shallower than 30 km that occurred between 1997 and 2024 and were located within 0.3° of a grid point with stress data (Fig. 2A).

For each earthquake, we compute the normal and shear stresses on the two nodal planes using the corresponding local stress tensor σ. Let $n_{1}$ and $n_{2}$ be the unit normal vectors of the two nodal planes. The normal stress on plane i is given by

$$\sigma_{n}=n_{i}\cdot \sigma \cdot n_{i}$$

This quantity can be negative, but it is assumed to become positive once unknown absolute stress components are added. There are two ways to compute shear stress. The maximum shear stress on the plane is

$$\tau_{s}=|\sigma \cdot n_{i}-\sigma_{n}n_{1}|$$

which is always positive. Alternatively, the shear stress in the slip direction is calculated using the event’s focal mechanism as

$$\tau_{e}=n_{1}\cdot \sigma \cdot s_{2}=n_{2}\cdot \sigma \cdot s_{1}$$

where $s_{1}$ and $s_{2}$ are the slip vectors on the respective planes. This quantity may be positive or negative; negative values indicate inconsistency with the background stress field, although such events are present in the catalog.

To quantify the consistency between a focal mechanism and the background stress, we define a metric based on the deviation from the Coulomb failure criterion. Let I be the inner product between the vector $\left[\sigma_{n},\tau_{e}\right]$ and the unit vector pointing toward the tangent point of the Coulomb failure criterion $\tau =\mu \sigma_{n}$ from the center of the Mohr circle, namely, $\left[-\text{cos}2\theta^{\text{opt}},\text{sin}2\theta^{\text{opt}}\right]$. This index ranges from −1 to 1 (Fig. 2B). Although slightly different in definition, this quantity is linearly related to the instability index $I_{v}$ defined by Vavryčuk (*23*) through

$$I_{v}=\text{cos}2\theta^{\text{opt}}+\left(1-\text{cos}2\theta^{\text{opt}}\right)I$$

and we refer to it as “instability” in this study as well.

We divide the events into five groups based on their I values and estimate the b value and its SE for each group (Fig. 2, D and E). The completeness magnitude $M_{c}$ was tested over the range of 4.2 to 4.5 to confirm that the results are stable.

### Global background stress and strike-slip earthquake mechanisms

The WSM (*25*) provides estimates of the orientation of the maximum horizontal compressive stress (*SH*_max_) at locations worldwide. These estimates are derived from various types of data, including earthquake focal mechanisms, borehole measurements, and geological indicators of crustal deformation, with earthquake data being the most dominant source. The stress direction at a given location is inferred from nearby observations within a variable search radius. Among the three catalogs provided by WSM, we used the one containing the largest number of estimates—specifically, the smoothed stress data obtained with a variable search radius of 100 to 1000 km on a 0.2° grid, distributed as “mean_SHmax_r1000_100_02.dat.” Analysis using the other two catalogs yielded fewer events and larger uncertainties but produced qualitatively similar results.

We then extracted strike-slip earthquakes from the GCMT catalog, limited to events shallower than 30 km and with both nodal planes dipping more than 60°. We selected only those events located within 0.3° of a grid point in the WSM dataset (fig. S4).

We defined the horizontal azimuthal difference Δϕ between the direction of *SH*_max_ and the *P* axis of the focal mechanism. This angle ranges from 0° to 90° (Fig. 3A). The events were grouped into 10 equal-sized bins based on Δϕ, and for each group, the b value and its SE were computed (Fig. 3, B and C).

### Derivation of Eq. 3

We consider the relationship between the $\sigma_{1}$ axis and dipping fault plane in subduction zones (fig. S8). Let θ be the angle between the fault plane and the direction of maximum compressive stress. The shear stress τ and normal stress $\sigma_{n}$ on the fault plane are given by Mohr’s circle relations

$$\tau =\frac{\sigma_{1}-\sigma_{3}}{2}\text{sin}2\theta =\frac{\text{Δσ}}{2}\text{sin}2\theta$$

$$\sigma_{n}=\frac{\sigma_{1}+\sigma_{3}}{2}-\frac{\sigma_{1}-\sigma_{3}}{2}\text{cos}2\theta =\sigma_{0}-\frac{\text{Δσ}}{2}\text{cos}2\theta$$

If the $\sigma_{1}$ axis is inclined by an angle δ below the horizontal, the vertical stress $\sigma_{\mathrm{zz}}$ corresponds to the normal stress on a plane tilted by δ

$$\sigma_{\mathrm{zz}}=\frac{\sigma_{1}+\sigma_{3}}{2}-\frac{\sigma_{1}-\sigma_{3}}{2}\text{cos}2\delta =\sigma_{0}-\frac{\text{Δσ}}{2}\text{cos}2\delta$$

Combining these equations with the Coulomb failure criterion (Eq. 2), we obtain

$$\text{Δσ}=\frac{2\left(\sigma_{\mathrm{zz}}-p\right)}{\mu^{-1}\text{sin}2\theta +\text{cos}2\theta -\text{cos}2\delta }$$

Assuming slip occurs at the optimal angle $\theta^{\text{opt}}$, then

$$\theta =\theta^{\text{opt}}=\frac{1}{2}\text{atan}\frac{1}{\mu }$$

Substituting this into the previous expression yields

$$\text{Δσ}=\frac{2\left(\sigma_{\mathrm{zz}}-p\right)}{\sqrt{1+\mu^{2}}/\mu -\text{cos}2\delta }$$

which corresponds to Eq. 3 in the main text.
