# Supplementary material for: Ultralow-angle faults produce giant earthquakes
Source: Sci Adv. 2026 Jul 1;12(27):eaee3921. doi: 10.1126/sciadv.aee3921 (PMC13322238; doi:10.1126/sciadv.aee3921)
Supplement: Supplementary file 1 — Figs. S1 to S8 [file sciadv.aee3921_sm.pdf]

Science Advances
AAAS

Supplementary Materials for

Ultralow-angle faults produce giant earthquakes

Satoshi Ide *et al.*

Corresponding author: Satoshi Ide, ide@eps.s.u-tokyo.ac.jp

Sci. Adv. **12**, eaee3921 (2026)
DOI: 10.1126/sciadv.aee3921

This PDF file includes:

Figs. S1 to S8

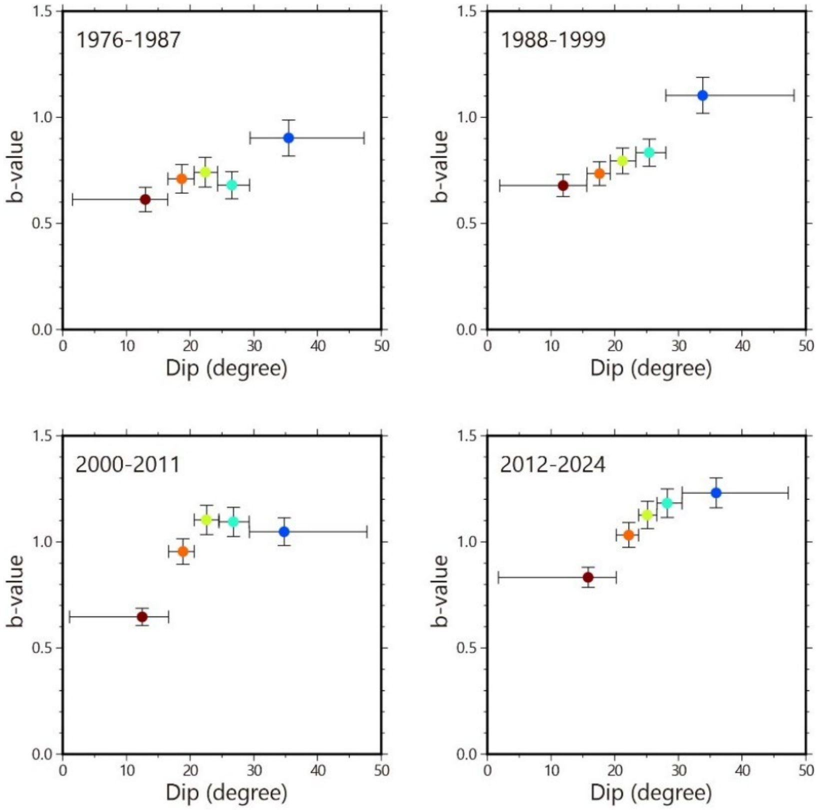

Fig. S1. Temporal variations in the relationship between dip angle and *b*-value. Same plots as Fig. 1D, using subcatalogs divided into four time periods. Each panel shows the time range (in years) used for the analysis in the upper left. For each subcatalog, earthquakes are grouped into five dip-angle bins (horizontal bars), and the corresponding *b*-values and their standard errors (vertical bars) are plotted.

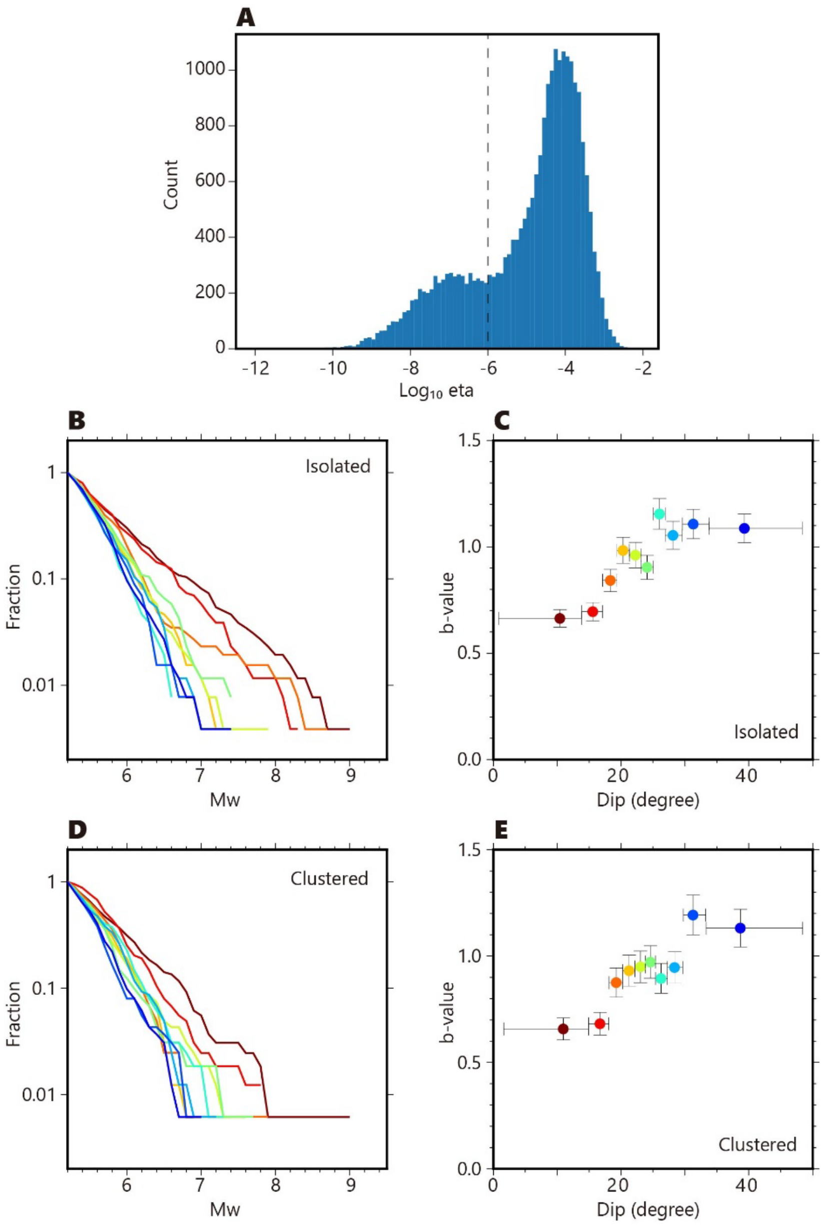

Fig. S2. Dip angle and *b*-value of reverse-fault earthquakes classified using the nearest-neighbor method*(24)*. (A) Histogram of $\log_{10} \eta$ calculated for all events in the GCMT catalog. The dashed line divides isolated and clustered events. **(B)** Magnitude–frequency distributions of isolated events for ten dip-angle bins, with colors corresponding to the ranges shown in panel **C**. **(C)** Relationship between dip angle and *b*-value for isolated events. Horizontal bars represent the dip range of each bin, and vertical bars indicate the standard error. **(D)** Same as **B**, but for clustered events. **(E)** Same as **C**, but for clustered events.

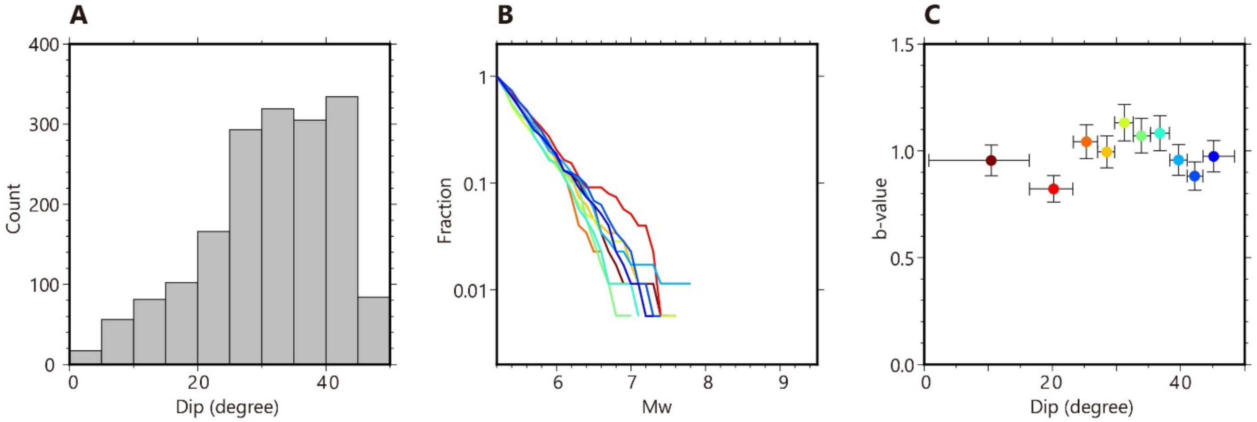

Fig. S3. Dip angle and *b*-value of deep reverse-fault earthquakes. Events are from the 1976–2024 GCMT catalog, deeper than 50 km and with $M_w > 5.2$, and classified as reverse faults (both nodal planes have rake angles between 60° and 120°). **(A)** Histogram of dip angles. **(B)** Magnitude–frequency distributions for ten dip-angle bins, with colors corresponding to the ranges shown in panel **C**. **(C)** Relationship between dip angle and *b*-value. Horizontal bars represent the dip range of each bin, and vertical bars indicate the standard error.

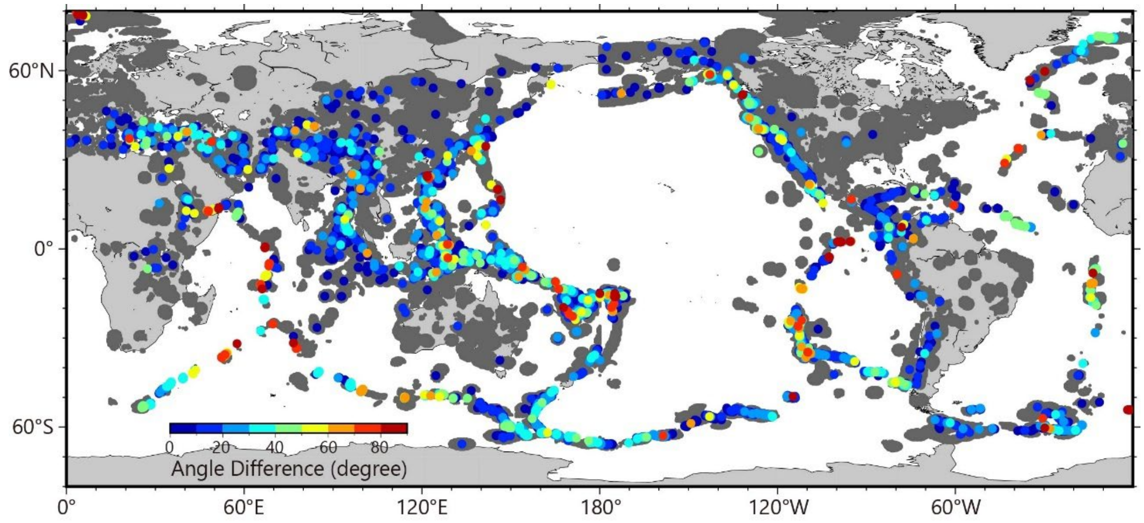

Fig. S4. Regions of estimated maximum horizontal stress and angular difference $\Delta\phi$ between GCMT P-axes and SH_{max}. Earthquakes used in Fig. 3A–C are shown with colors indicating $\Delta\phi$, the angle between the GCMT P-axis and the estimated direction of maximum horizontal compressive stress (SH_{max}). Gray dots mark locations where SH_{max} estimates are available from the WSM.

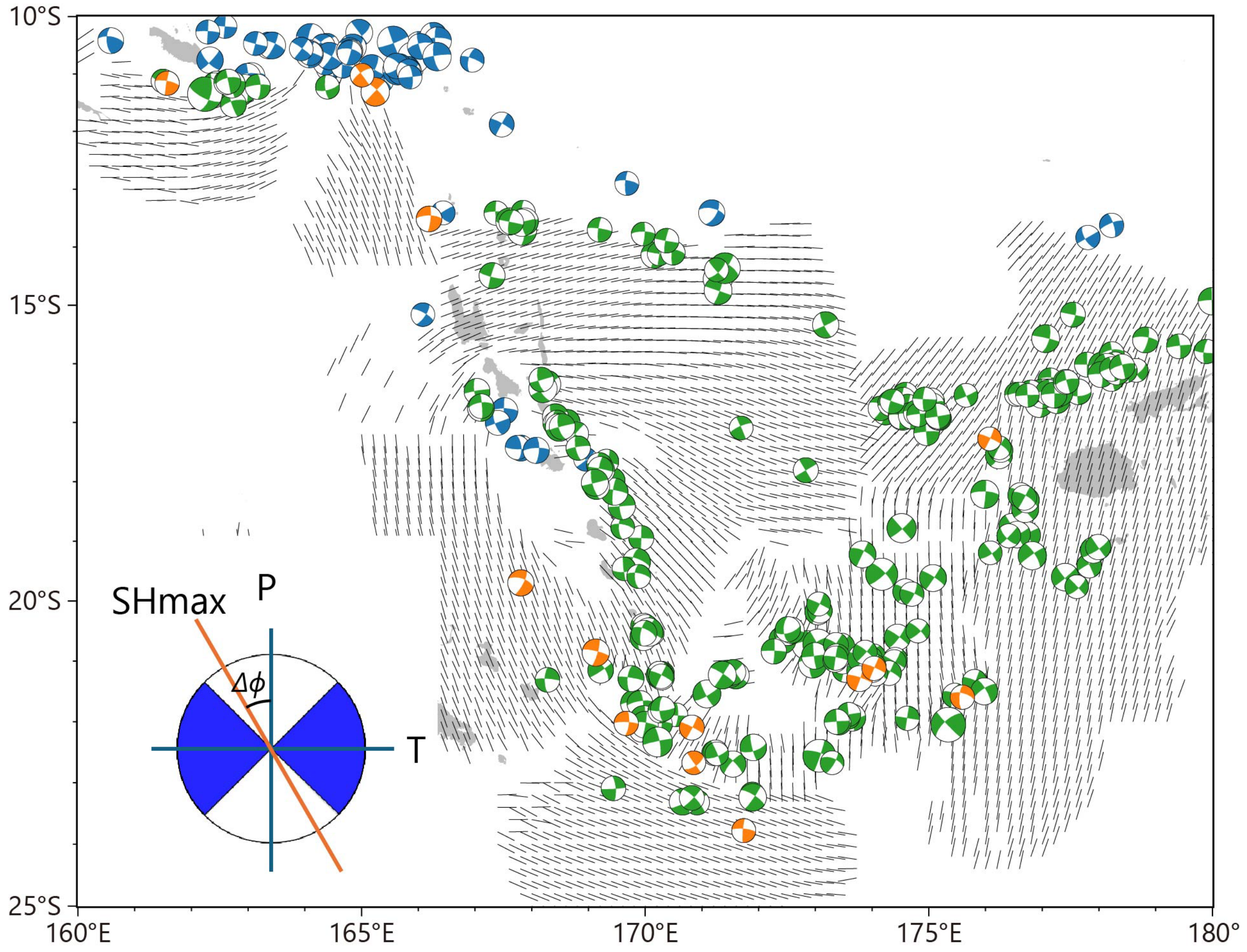

Fig. S5. Examples of WSM stress fields and GCMT focal mechanisms. The definition of the angular difference $\Delta\phi$ is illustrated in the lower left. Blue symbols indicate earthquakes not used in the analysis; green symbols represent events with $\Delta\phi < 45°$, and orange symbols represent those with $\Delta\phi \geq 45°$.

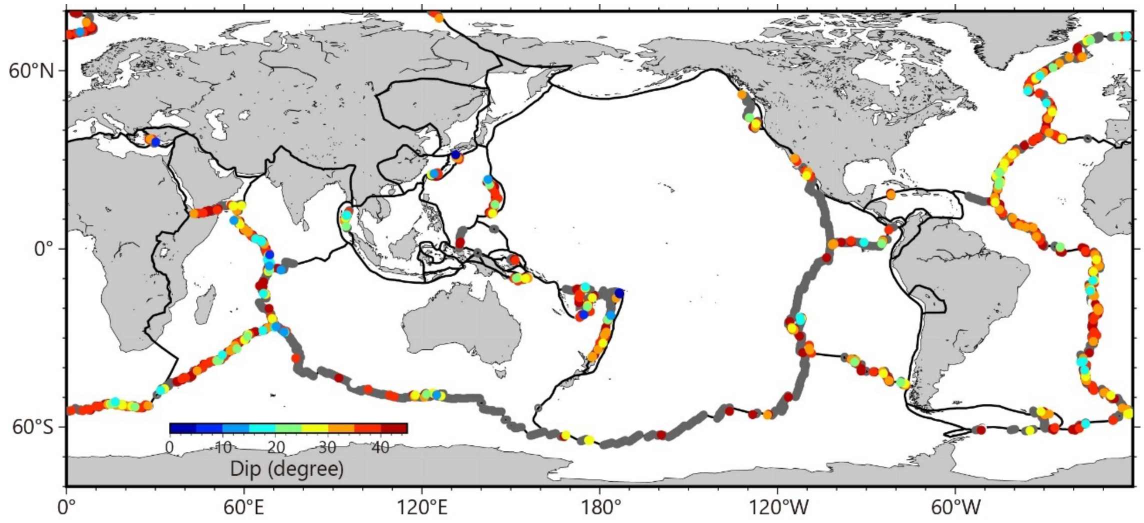

Fig. S6. Distribution of plate boundaries and normal faulting earthquakes. Plate boundaries from PB2002 (*19*) are shown as black lines, with spreading axes highlighted by thick gray lines. Earthquakes used to generate Fig. 3D–F are color-coded by dip angle.

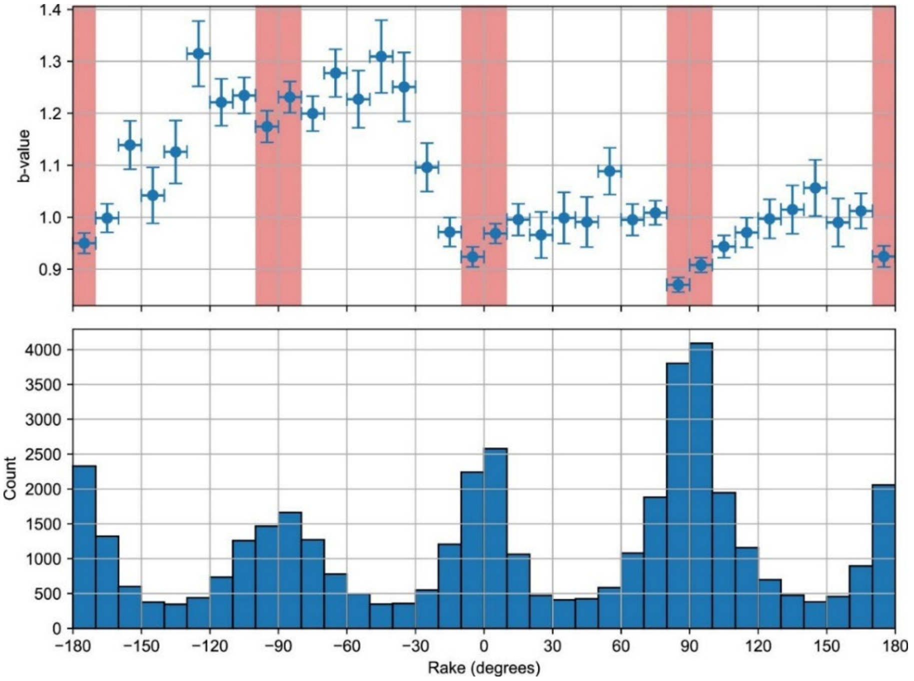

Fig. S7. Relationship between rake angle and *b*-value for GCMT earthquakes. For earthquakes shallower than 30 km and with $M_w > 5.2$ in the GCMT catalogue, *b*-values were calculated in 10° intervals of rake angle. Each earthquake contributes twice—once for each nodal plane. (Top) *b*-value versus rake angle. Red shading highlights the ±10° ranges around rake angles predicted by Anderson's theory. (Bottom) Corresponding histogram of earthquake counts.

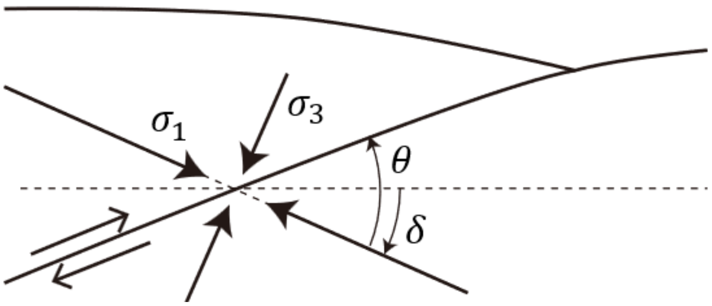

Fig. S8. Definition of the plunge angle of the σ_1-axis (δ) and the angle between to the σ_1-axis and the plate interface (θ).
